# FAM20A is a golgi-localized Type II transmembrane protein

**DOI:** 10.1038/s41598-024-57007-z

**Published:** 2024-03-18

**Authors:** Mohammad Faizan Siddiqui, Jiahe Li, Suzhen Wang, Hua Zhang, Chunlin Qin, Yongbo Lu

**Affiliations:** Department of Biomedical Sciences, Texas A&M University School of Dentistry, 3302 Gaston Ave, Dallas, TX 75246 USA

**Keywords:** Biochemistry, Cell biology, Molecular biology

## Abstract

Family with sequence similarity 20, member A (FAM20A) is a pseudo-kinase in the secretory pathway and is essential for enamel formation in humans. Here we examine if FAM20A is a membrane-associated protein. We show that the full-length FAM20A can be purified from HEK293 cells transfected with a FAM20A-expresing construct. Further, it is only found in the membrane fraction, but not in the soluble fraction, of cell lysate. Consistently, it is not secreted out of the expressing cells. Moreover, it is co-localized with GM130, a cis-Golgi network marker, and membrane topology analysis indicates that it has its C-terminus oriented towards the lumen of the organelle. Our results support that FAM20A is a Type II transmembrane protein within the secretory compartments.

## Introduction

Family with sequence similarity 20 (FAM20) family consists of three members of highly related proteins in mammals, including FAM20A, FAM20B and FAM20C. FAM20A was originally discovered in mouse hematopoietic cells^1^, and was later considered as a pseudo-kinase within the secretory pathway as it lacks a crucial residue required for catalysis^2^. It forms a complex with FAM20C and allosterically enhances the kinase activity of the latter in phosphorylating secretory proteins, including enamel matrix proteins and small integrin-binding ligand N-linked glycoproteins (SIBLINGs)^2–4^. The phosphorylation of these proteins is essential for the development and mineralization of enamel, dentin and bone. FAM20B is a xylose kinase required for biosynthesis of glycosaminoglycan chains of chondroitin sulfate and heparan sulfate proteoglycans^5^.

Human genetic and animal studies also suggest a potential functional interaction between FAM20A and FAM20C. During tooth development, *Fam20a* and *Fam20c* are co-expressed in enamel-forming ameloblasts as well as dentin-forming odontoblast^6–8^. Mutations in the *FAM20A* gene in humans cause autosomal recessive hypoplastic amelogenesis imperfecta (AI)^9–11^. Likewise, *Fam20a* deletion in ameloblasts in mice led to a tooth phenotype that resembles human AI associated with *FAM20A* mutations. It is characterized by poorly differentiated ameloblasts and development of cystic lesions in the mandibular molars^6,12,13^. A similar enamel phenotype has been observed in *Fam20c*-deficient mice^13–15^.

More recently, FAM20C has been found to be a Type II transmembrane protein within the secretory compartments, with its N-terminal signal peptide-like region serving as a membrane anchor for Golgi retention^16^. However, it is unclear whether FAM20A functions as a membrane protein or soluble protein that interacts with FAM20C.

In this study, we purified FAM20A from the cells transfected with a construct expressing FAM20A attached with a histidine tag, and confirmed the purification of the full-length FAM20A by mass spectrometry. Moreover, we demonstrated that FAM20A was predominantly present in the membrane fraction but was not secreted out of the expressing cells. By co-immunofluorescent staining, we further showed that FAM20A was primarily localized within the cis-Golgi network in the odontoblast-like cells and ameloblast-like cells in vitro. Moreover, we found that FAM20A was localized in the cis-Golgi network in odontoblasts and ameloblasts in vivo. In addition, membrane topology analysis showed that FAM20A had its C-terminus localized within the lumen of the intracellular organelles. These findings support that FAM20A exists as a Type II transmembrane protein within the secretory compartments.

## Results

### Purification and MS analysis of FAM20A-HIS

**Figure 1:**
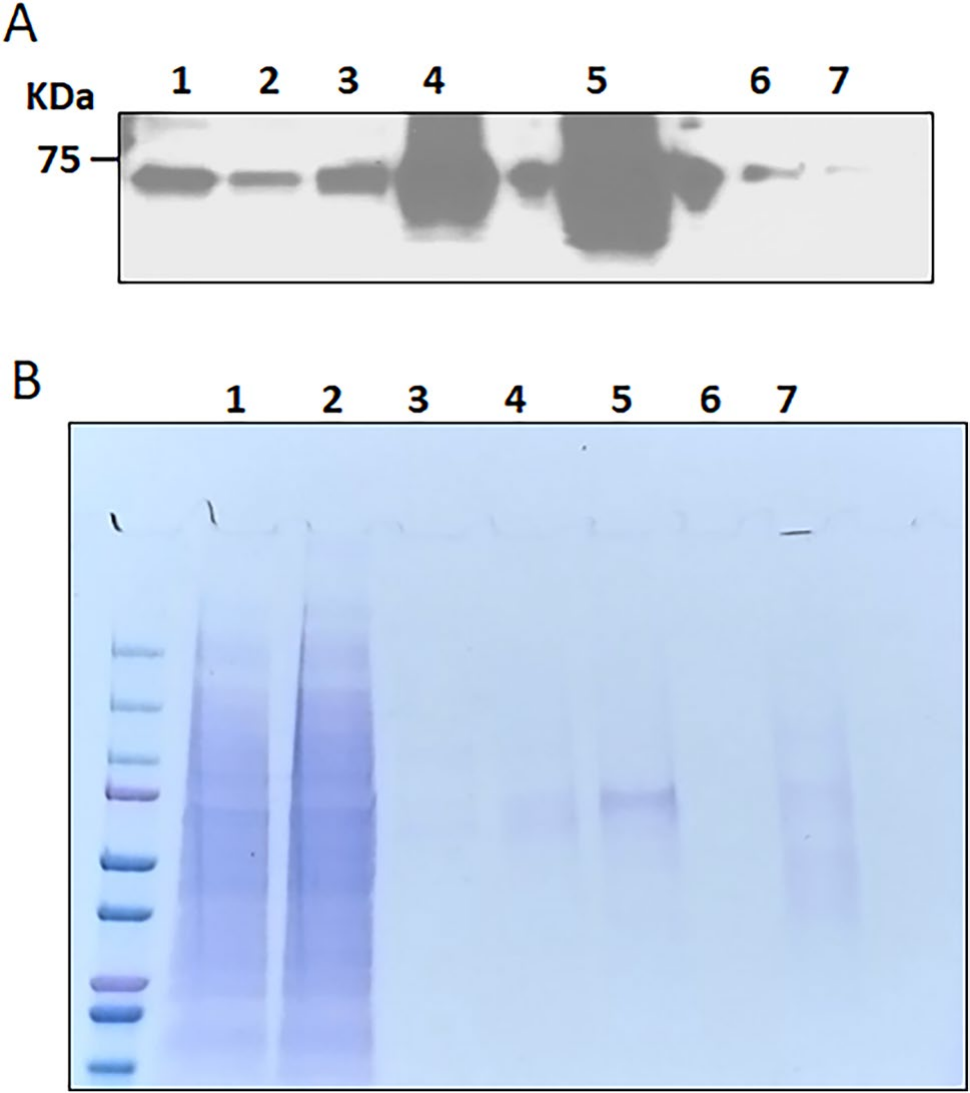
Western-blotting and Coomassie blue staining analyses of purified FAM20A. The fractions eluted from IMAC were analyzed by Western-blotting analysis using an anti-HIS antibody for detection of FAM20A-HIS (**A**), and by Coomassie blue staining of SDS-PAGE gel (**B**). Lane 1 is the cell lysate; lane 2 is the flow-through; and lanes 3 through 7 are the fractions eluted with 50, 100, 250, 500, and 1000 mM imidazole, respectively. The original blot and gel are provided in Supplementary Fig. S1.

**Figure 2:**
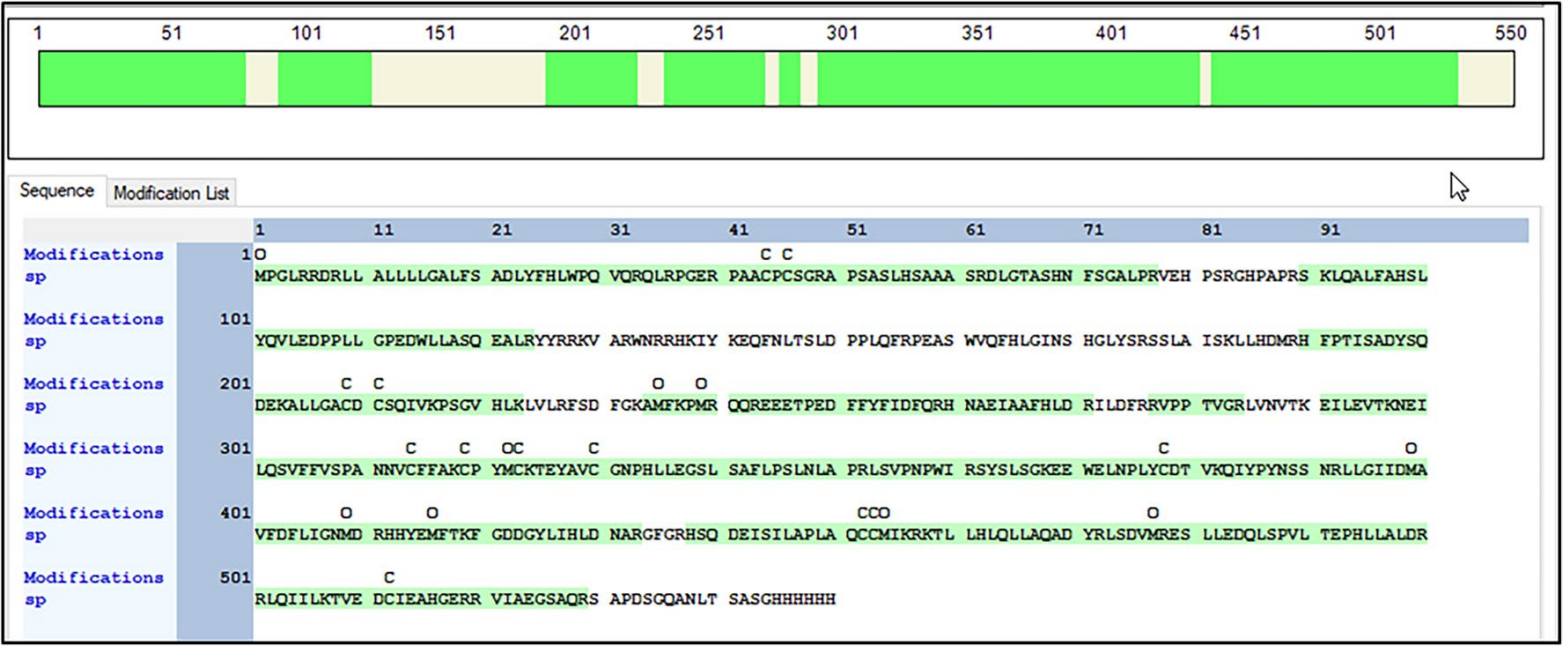
Mass spectrometric analysis of FAM20A purified by IMAC. The FAM20A gel band was excised from the Coomassie blue stained SDS-PAGE gel and subjected to tryptic digestion followed by mass spectrometric analysis. Raw mass spectrometric data files were analyzed using Proteome Discoverer v2.4 SP1, with peptide identification performed using Sequest HT searching against the human protein database from UniProt. The protein in the band shows a coverage of 77.64% of the amino acid sequence of FAM20A. The peptides identified by mass spectrometry from tryptic digests of FAM20A gel band are presented in Supplementary Table 1.

Ni–NTA column tends to bind HIS tag attached to either ends of a protein and helps in isolation of the protein of interest. Hence, HEK293 cells were transiently transfected with the construct expressing FAM20A-HIS, and total cell lysate was harvested using RIPA buffer 48 h after transfection. The lysate was centrifuged, and supernatant was loaded on Ni–NTA column. The flow-through was collected and the column was then washed with washing buffer containing 20 mM imidazole to remove the proteins that bound nonspecifically to the column. Finally, FAM20A-HIS was eluted using different concentrations of imidazole. Western-blotting analysis with a mouse monoclonal anti-HIS antibody shows the presence of a protein band corresponding to FAM20A-HIS in the lysate, flow-through, and different fractions eluted with 50, 100, 250, 500, and 1000 mM imidazole, respectively (Fig. 1A). It is evident that the amount of FAM20A-HIS is highest in 250 mM imidazole elution fraction (Fig. 1A, lane 5). Coomassie blue staining of SDS-PAGE gel was also performed to check the purity of FAM20A-HIS in different imidazole elution fractions (Fig. 1B). Again, a single prominent band that corresponds to FAM20A-HIS is observed in 250 mM imidazole elution fraction (Fig. 1B, lane 5). However, no distinct bands are found in other lanes, suggesting that the amount of FAM20A-HIS in other elution fractions is too low to be detected by Coomassie blue staining. The single protein band was accurately excised from lane 5 on the Coomassie blue stained gel, followed by digestion with trypsin for MS analysis to determine if the full-length FAM20A was purified. Figure 2 shows the sequencing data processed using Proteome Discoverer 2.4. It is evident that the protein present in the band matches with the sequence of FAM20A with a coverage of 77.64%. Peptides identified by mass spectrometry from the tryptic digests of FAM20A gel band are shown in Supplementary Table 1 online. It is of note that MS analysis reveals the intact N terminal amino acid sequence of FAM20A. Taken together, these results strongly demonstrate the purification of the full-length FAM20A-HIS from the FAM20A-HIS-expressing HEK293 cells.

### Membrane localization of FAM20A

**Figure 3:**
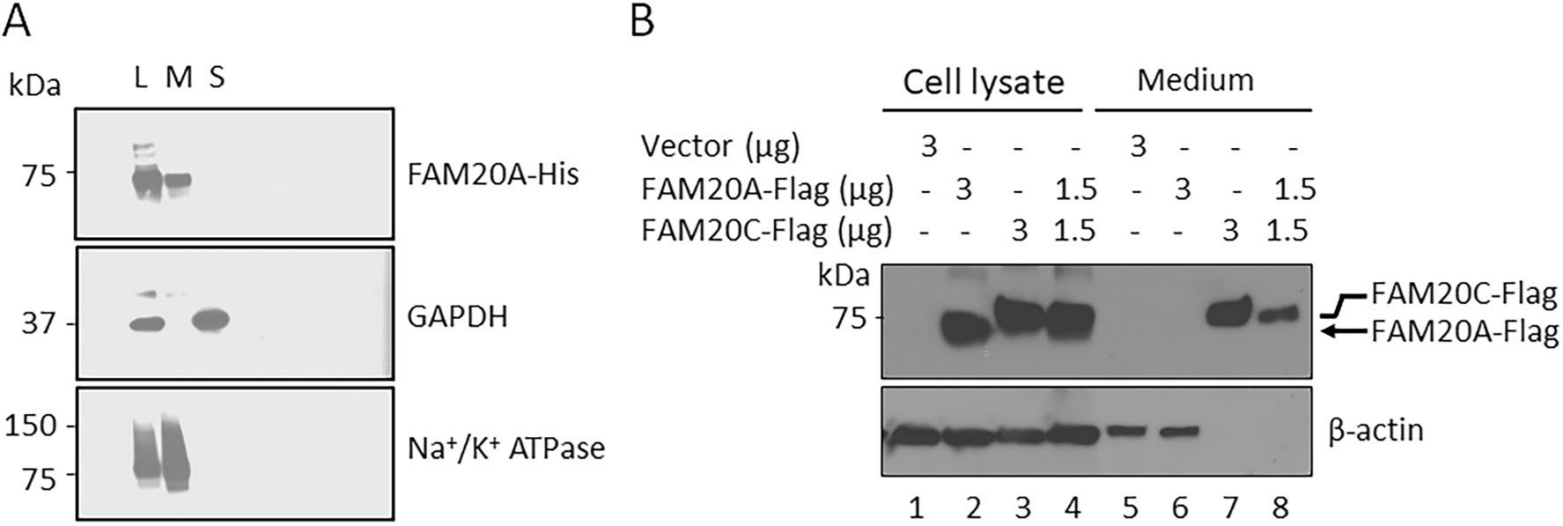
Membrane localization of FAM20A. (**A**) FAM20A present in the membrane fraction of the expressing cells. Western-blotting analysis of FAM20A in the membrane and soluble fractions extracted from HEK293 cells transiently transfected with the construct expressing FAM20A-HIS. The blot was first probed with an anti-HIS antibody against FAM20A-HIS. The blot was then sequentially probed with an anti-GAPDH and anti-sodium potassium (Na^+^K^+^) ATPase antibody. GAPDH and sodium potassium ATPase were used as a cytosolic protein marker and plasma membrane protein marker, respectively. L, total cell lysate; M, membrane fraction; and S, soluble fraction. (**B**) FAM20A is not secreted out the expressing cells. HEK293 cells were transiently transfected with pCDNA3 empty vector (Vector) or a construct expressing FAM20A-FLAG and/or FAM20C-FLAG. The total cell lysates and conditioned media were harvested and analyzed by Western-blotting with a mouse monoclonal anti-FLAG M2 antibody, and the blot was then stripped and probed with a mouse monoclonal β-actin antibody. The amount of DNA transfected for each construct are indicated. For lanes 1, 2 and 3, 60 µg of total cell lysates were loaded. For lanes 5 and 6, 1 ml of conditioned medium was loaded after concentration; and for lanes 7 and 8, 20 µl of conditioned medium was loaded. Note that β-actin was detectable in lanes 5 and 6 loaded with concentrated medium. The original blots are provided in Supplementary Fig. S2.

To further determine whether FAM20A is a membrane or soluble protein, we performed total membrane fractionation using Mem-PER™ Plus membrane protein extraction kit. HEK293 cells transfected with the construct expressing FAM20A-HIS were lysed either by RIPA buffer to extract total cell lysate, or were subjected to cell permeabilization buffer for extraction of soluble proteins followed by solubilization of the pellet in membrane solubilization buffer to extract membrane proteins. The total cell lysate, membrane fraction and soluble fraction were analyzed by Western-blotting analysis with an anti-HIS antibody to detect FAM20A-HIS in respective preparation (Fig. 3A, top). The blot was then stripped and sequentially probed with an antibody against GAPDH (Fig. 3A, middle) and an antibody that recognizes sodium potassium ATPase (Fig. 3A, bottom). GAPDH is used as a cytosolic marker and sodium potassium ATPase is used as a plasma membrane marker^17,18^. As expected, FAM20A-HIS, GAPDH and sodium potassium ATPase are all present in the total cell lysate (Fig. 3A, lane L). However, it is evident that FAM20A-HIS is present only in the membrane fraction (Fig. 3A, lane M), but not in the soluble fraction (Fig. 3A, lane S). Moreover, GAPDH is not detected in the membrane fraction (Fig. 3A, lane M), whereas sodium potassium ATPase is not found in the soluble fraction (Fig. 3A, lane S), thereby excluding cross contamination of the membrane fraction with the soluble fraction, and vice-versa. These results indicate that FAM20A is a membrane protein. Moreover, we transfected a construct expressing FAM20A-FLAG and/or FAM20C-FLAG into HEK293 cells, and harvested the total cell lysates and conditioned medium 48 h after transfection. Western-blotting analysis showed that FAM20A-FLAG was only detected in the cell lysate but not in the conditioned medium (Fig. 3B lane 2 and 6). Unlike FAM20A, FAM20C was not only detected in the cell lysate, but also was readily detected in the conditioned medium (Fig. 3B lane 3 and 7). In addition, co-transfection of the FAM20C-FLAG-expressing construct did not help the secretion of FAM20A-FLAG (Fig. 3B lane 4 and 8). Collectively, these results support that FAM20A is a membrane-associated protein and is not secreted out of the expressing cells.

### Golgi-localization of FAM20A

**Figure 4:**
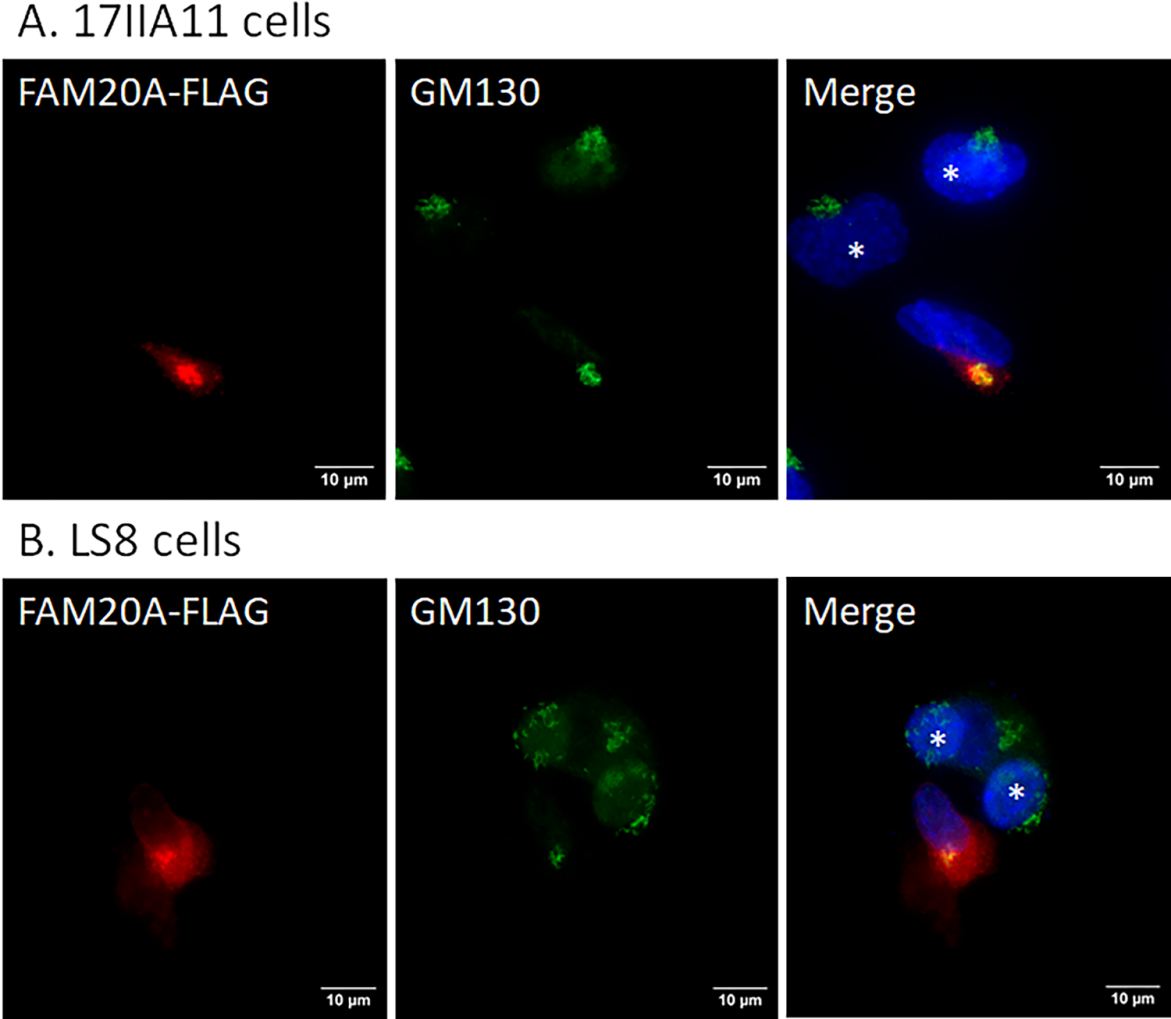
Subcellular localization of exogenous FAM20A in 17IIA11 and LS8 cells. 17IIA11 odontoblast-like cells (**A**) and LS8 ameloblast-like cells (**B**) were transiently transfected with the FAM20A-FLAG construct, and were then permeabilized with 0.1% Triton X-100 and immunofluorescently labeled with a mouse monoclonal anti-FLAG M2 antibody that detects FAM20A-FLAG (signal in red) and a rabbit anti-GM130 antibody (signal in green). Nuclei were stained with DAPI (blue). GM130 is a marker for the cis-Golgi network. The merged images show co-localization (yellow) of FAM20A-FLAG and GM130. *Marks the non-transfected cells. Scale bars: 10 µm.

**Figure 5:**
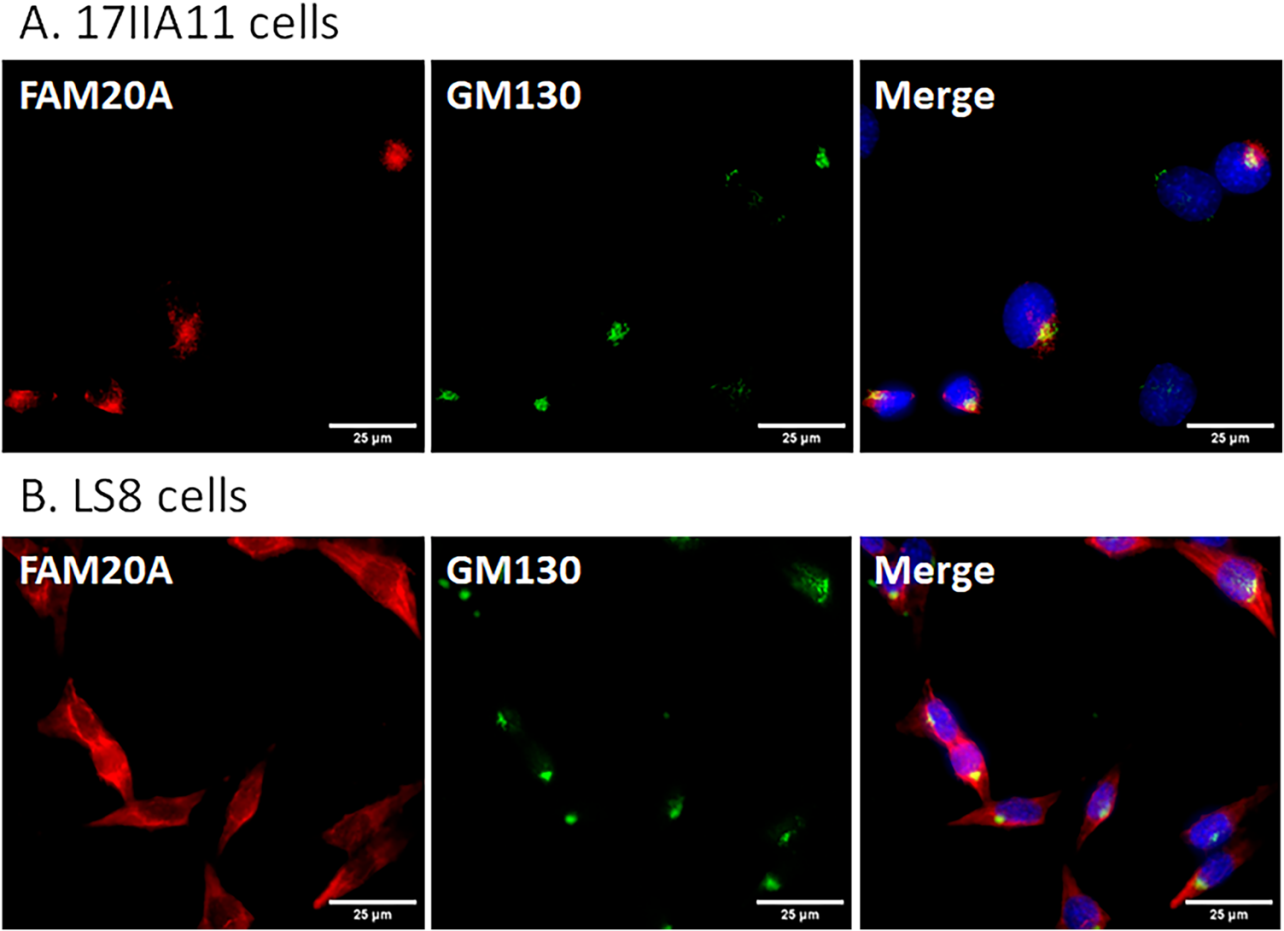
Subcellular localization of endogenous FAM20A in 17IIA 11 and LS8 cells. Non-transfected 17IIA11 cells (**A**) and LS8 cells (**B**) were permeabilized with 0.1% Triton X-100 and immunofluorescently labeled with a rabbit anti-FAM20A antibody that detects endogenous FAM20A (signal in red) and a mouse monoclonal anti-GM130 antibody (signal in green). Nuclei were stained with DAPI (blue). GM130 is a marker for the cis-Golgi network. The merged images show co-localization (yellow) of FAM20A and GM130. Scale bars: 25 µm.

**Figure 6:**
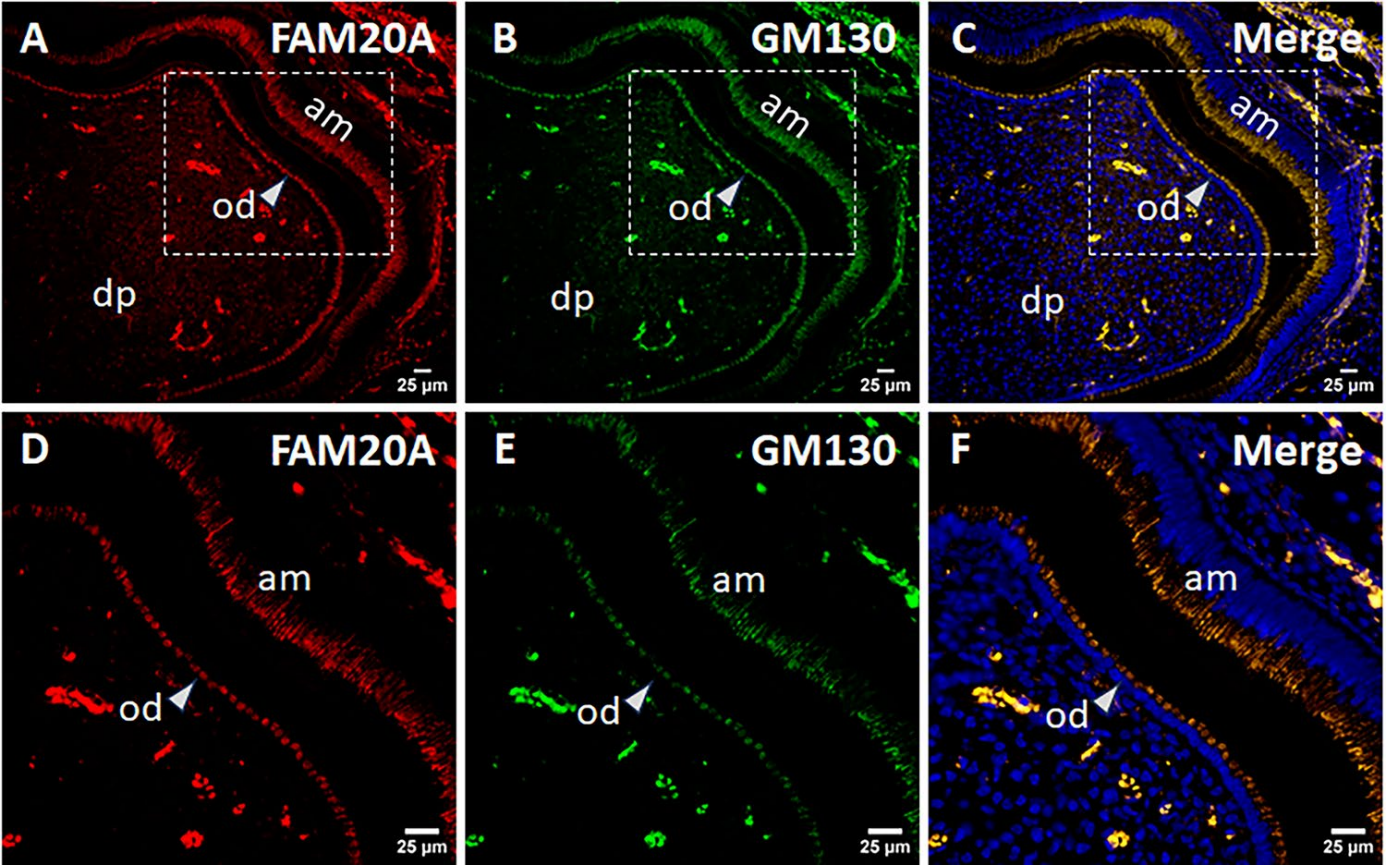
Subcellular localization of FAM20A in odontoblasts and ameloblasts in mice. Shown are representative images of immunohistochemical results of a sagittally-sectioned mandibular first molar of 4-day-old wild-type mice. The tissue section was immunofluorescently labeled with a rabbit polyclonal anti-FAM20A antibody that detects endogenous FAM20A (signal in red) (**A**,**D**) and a mouse monoclonal anti-GM130 antibody (signal in green) (**B**,**E**). Nuclei were stained with DAPI (blue) (**C**,**F**). GM130 is a marker for the cis-Golgi network. The merged image shows co-localization (yellow) of FAM20A and GM130 in odontoblasts and ameloblasts (**C**,**F**). (**D**–**F**) are the higher magnification views of the boxed area in (**A**–**C)**, respectively. od, odontoblasts; am, ameloblasts; and dp, dental pulp. Scale bars: 25 µm.

We further investigated the subcellular localization of FAM20A in 17IIA11 odontoblast-like cells and LS8 ameloblast-like cells and in odontoblasts and ameloblasts in mice. First, 17IIA11 and LS8 cells transiently transfected with the construct expressing FAM20A-FLAG were incubated with a mouse monoclonal anti-FLAG M2 antibody against FAM20A-FLAG (signal in red; Fig. 4A,B), and a rabbit polyclonal antibody that recognizes GM130 which is cis-Golgi-localized peripheral membrane protein^19^ (signal in green; Fig. 4A,B). The nuclei were stained with DAPI (signal in blue; Fig. 4A,B). The merged images clearly show that the spot with intense FAM20A-FLAG signals was co-localized with GM130 signals in 17IIA11 cells (Fig. 4A) and in LS8 cells (Fig. 4B). Further, we performed co-immunofluorescent staining with a rabbit polyclonal anti-FAM20A antibody that detects endogenous FAM20A (signal in red) and a mouse monoclonal anti-GM130 antibody (signal in green), and showed that endogenous FAM20A was also co-localized with GM130 in 17IIA11 cells and LS8 cells (Fig. 5). Moreover, immunohistochemistry with the same rabbit polyclonal anti-FAM20A antibody and mouse monoclonal anti-GM130 antibody revealed that FAM20A was co-localized with GM130 in odontoblasts and ameloblasts in the mandibular first molars of 4-day-old wild-type mice (Fig. 6). Altogether, these findings demonstrate that FAM20A is primarily localized to the cis-Golgi network in odontoblast-like cells and ameloblast-like cells in vitro as well as in odontoblasts and ameloblasts in vivo.

### Membrane topology of FAM20A

**Figure 7:**
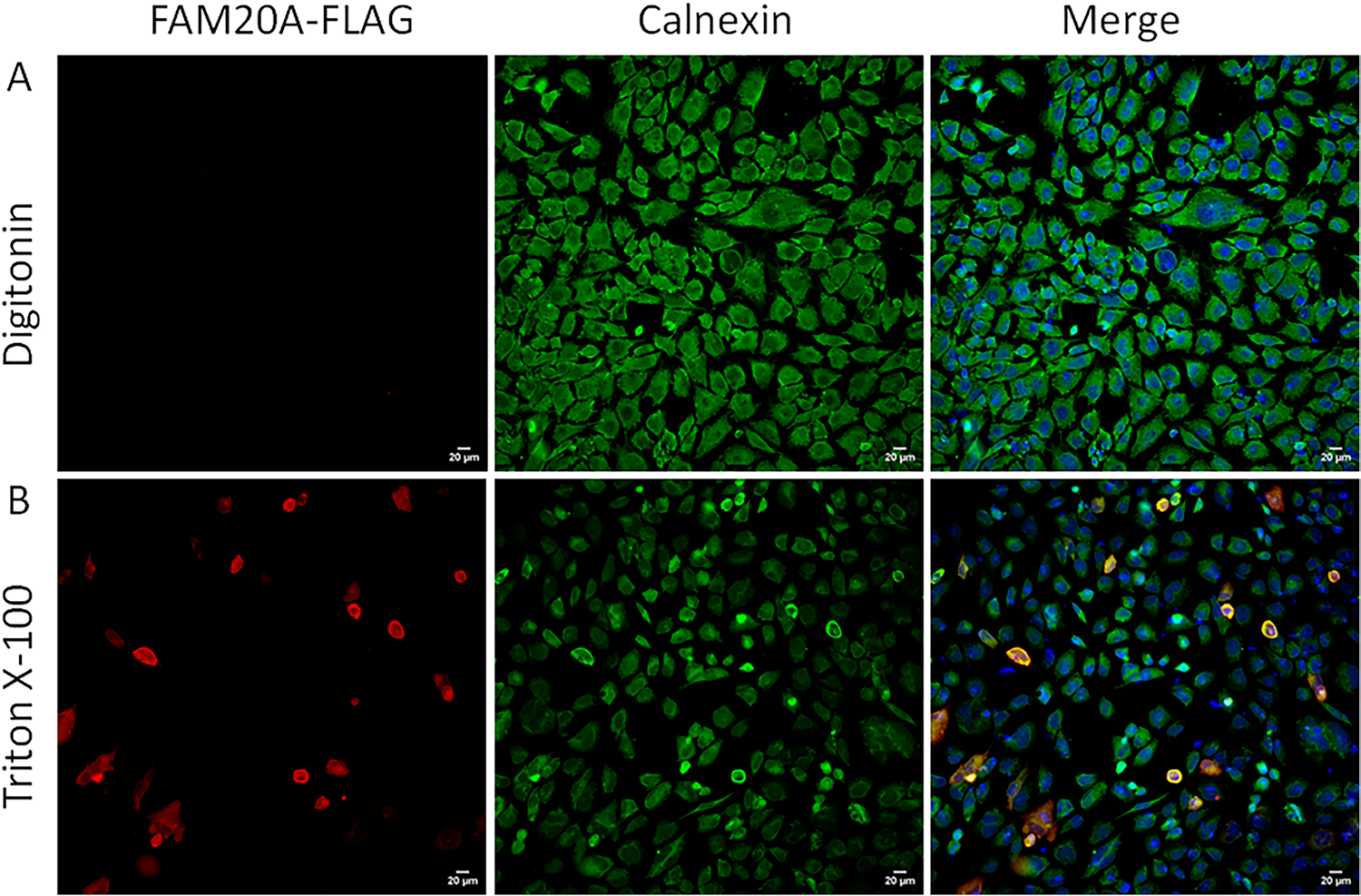
Membrane topology of FAM20A-FLAG. 17IIA11 odontoblast-like cells were transiently transfected with the FAM20A-FLAG construct, and the cells were then treated with either 12.5 µM digitonin (**A**) or 0.1% Triton X-100 (**B**). The cells were immunofluorescently labeled with an anti-FLAG M2 antibody against FAM20A-FLAG (signal in red) and an antibody against the cytosolic domain of Calnexin (signal in green). Calnexin is an ER-localized transmembrane protein. Nuclei were stained with DAPI (blue). Scale bar 20 µm.

Digitonin is a nonionic detergent that has an affinity for cholesterol that is present abundantly in the plasma membrane than in the membranes of other intracellular organelles^20^. It is frequently used to selectively permeate the plasma membrane but not the membranes of the endoplasmic reticulum (ER) or other organelles^21^. In contrast, Triton X-100 is a nonionic surfactant that is widely used to permeabilize all the membranes of living cells, including intracellular organelle membranes^22^. To determine the membrane topology of FAM20A, 17IIA11 cells transfected with the FAM20A-FLAG construct were permeabilized with either 12.5 µM digitonin (Fig. 7A) or 0.1% Triton X-100 (Fig. 7B). The cells were then incubated with an anti-FLAG M2 antibody against FAM20A-FLAG and an antibody that recognizes the cytosolic domain of transmembrane protein calnexin, an ER resident protein^23^. As expected, the signals for calnexin (signal in green) were observed in the cells treated with either digitonin (Fig. 7A) or Triton X-100 (Fig. 7B). However, the signals for FAM20A-FLAG (signal in red) were only detected in the cells permeabilized with Triton X-100 (Fig. 7B), but not in the cells treated with digitonin (Fig. 7A). These results indicate that the C-terminus of FAM20A-FLAG is oriented towards the lumen of the organelles, as the FLAG tag is appended to the C-terminus of FAM20A.

## Discussion

FAM20A is a member of FAM20 family of highly related proteins, and is essential for enamel formation during tooth development. In this study, we employed a combination of molecular and cellular approaches and studied the amino acid sequence, secretion, subcellular localization and membrane topology of FAM20A. We demonstrated that FAM20A existed as a full-length protein in HEK293 cells transfected with a construct expressing FAM20A-HIS, as evidenced by mass spectrometric analysis. We also showed that FAM20A was enriched in the membrane fraction and was not secreted when expressed in HEK293 cells. We presented that exogenous as well as endogenous FAM20A was primarily localized in the cis-Golgi network in 17IIA11 odontoblast-like cells and in LS8 ameloblast-like cells. Moreover, we showed that FAM20A was localized in the cis-Golgi network in odontoblasts and ameloblasts in mice. Our studies also documented that FAM20A had its C-terminus oriented towards the lumen of the organelles. These findings corroborate that FAM20A is a Type II transmembrane protein within the secretory compartments.

It has been controversial whether FAM20A can be secreted out of the expressing cells. When first discovered in hematopoietic cells, FAM20A was detected in both cell lysate and medium when the monkey kidney COS-1 cells were transfected with a construct expressing the full-length FAM20A^1^. Therefore, it was concluded that FAM20A was a secreted protein. Consistently, a signal sequence for entry into the secretory pathway was identified as the first 23 amino acids of FAM20A, such sequence was required for FAM20A secretion^1^. Later, FAM20A was also detected in both cell extract and medium of human osteosarcoma U2OS cells expressing FAM20A-FLAG^4^. Nevertheless, in our study, we were not able to detect FAM20A in the medium collected from HEK293 cells transfected with the FAM20A-FLAG construct. Consistent with our study, Ishikawa et al. reported that FAM20A was only detected in the cell lysate but not in the medium when HEK293T cells are transfected with a FAM20A-expressing construct^3^. The reason that accounts for the discrepancy between these studies is unknown and may be attributed to the difference in the cell lines used.

Accumulating evidence support that all FAM20 family members, including FAM20A, FAM20B and FAM20C, are a Type II membrane protein. First, it has been experimentally demonstrated that FAM20C is a Type II transmembrane protein within the secretory compartments^16^. Second, in this study, we have shown that FAM20A is a membrane protein primarily localized within the cis-Golgi network and presents a Type II transmembrane topology. Third, all members of the FAM20 family share a high degree of amino acid sequence similarity^1^. Lastly, membrane topology analysis done by ‘TOPCONS’^24^ shows that the N-terminal amino sequences of FAM20A (See Supplementary Fig. S3), FAM20B (See Supplementary Fig. S4) and FAM20C (See Supplementary Fig. S5) may serve as a signal peptide but also contains a transmembrane helix as predicted by different algorithms, suggesting that they have the potential to function as a signal/anchor sequence. Such signal/anchor sequence is not cleaved off by signal peptidase and is responsible for the entry of the respective protein into the ER as well as for subsequent anchorage of the protein to the ER membrane as a Type II transmembrane protein^25^. It has been thought that the signal/anchor sequence is essential for retention of Golgi-resident proteins^26–29^. Thereby, it remains to be studied whether the signal/anchor sequence is sufficient for retention of FAM20A within the Golgi complex.

In summary, we have demonstrated that FAM20A is a Type II transmembrane protein primarily localized in the cis-Golgi network. The new findings provide further evidence to support that membrane association may be a common feature of the FAM20 family members.

## Materials and methods

### Animals

All mice used in this study were on a C57BL/6 background and were bred and maintained in community housing (≤ 4 mice/cage, 22 °C) on a 12 h light/dark cycle with free access to water and standard pelleted food. All animal protocols were approved by the Institutional Animal Care and Use Committee (IACUC) of Texas A&M University (Dallas, TX) and were conducted in compliance with the ARRIVE guidelines. All animal procedures were performed in accordance with university and U.S. Public Health Service (PHS) Policy on Humane Care and Use of Laboratory Animals.

### DNA constructs

Two DNA constructs were generated to express mouse FAM20A in mammalian cells: one expressing FAM20A with a FLAG tag attached to the C-terminus (FAM20A-FLAG), and the other expressing FAM20A with a histidine (HIS) tag appended to the C-terminus (FAM20A-HIS). An additional DNA construct was generated to express mouse FAM20C with a FLAG tag fused to the C-terminus (FAM20C-FLAG). The vector used for these constructs is the pCDNA3 vector (Invitrogen). The three constructs were generated by polymerase chain reaction (PCR) or by site-directed mutagenesis using the QuikChange II XL site-directed mutagenesis kit (Cat. No. 200521; Agilent Technologies, Inc., Santa Clara, CA). All constructs were confirmed by enzymatic digestion and DNA sequencing.

### Cell culture and DNA transfection

HEK293 EBNA cells^30^, 17IIA11 odontoblast-like cells^31^ and LS8 ameloblast-like cells^32^ were grown and maintained in Dulbecco’s modified Eagle’s medium (DMEM) (Cat. No. 10-013-CV; Corning, USA) supplemented with 10% heat inactivated fetal bovine serum (FBS) (Cat. No. F2442; Sigma, USA), GlutaMAX (Cat. No. 35050061; Gibco, USA) and penicillin/streptomycin (Cat. No. 15070063; Gibco, USA) at 37 °C with 5% CO_2_ and 95% humidity. All cells were maintained in the logarithmic phase of growth and sub-cultured at 2 ± 3 days intervals. All DNA transfections were performed using X-tremeGENE™ 9 transfection reagent (Cat. No. 06365809001; Roche, Mannheim, Germany), according to the manufacturer’s instructions.

### Purification of FAM20A-HIS protein

Forty-eight hours after transfection with a construct expressing FAM20A-HIS, HEK293 cells were washed with PBS and lysed using radioimmunoprecipitation assay (RIPA) buffer containing cOmplete™, Mini, EDTA-free protease inhibitor cocktail (Cat. No. 11836170001; Roche, Mannheim, Germany), as previously described (Lu et al., Cells Tissues Organs, 2009). The cell lysate was centrifuged at 14,000 rpm for 20 min and supernatant was collected. The protein concentration was quantified using Pierce™ BCA protein assay kit (Cat. No. 23228; ThermoFisher Scientific).

HisPur™ Ni–NTA columns (Cat. No. 88225; ThermoFisher Scientific) were used for immobilized metal affinity chromatography (IMAC) for purification of FAM20A-HIS. Briefly, the supernatant containing FAM20A-HIS was loaded on Ni–NTA column pre-equilibrated with equilibration buffer containing 50 mM Tris–HCl (pH 7.5 at RT), 300 mM NaCl and 0.01% NaN_3_. The column was washed with 5 column volume of wash buffer containing 50 mM Tris–HCl (pH 8 at RT), 300 mM NaCl, 20 mM imidazole and 0.01% NaN_3_. The elution was done using different concentrations of imidazole (50, 100, 250, 500 and 1000 mM) in elution buffer. Each eluant was dialyzed and concentrated for downstream analyses.

### Western-blotting and Coomassie blue staining analyses of elution fractions

The eluants were run on 4–15% Mini-PROTEAN® TGX™ precast polyacrylamide gel electrophoresis (PAGE) gels (Cat. No. 4561083; Bio-Rad), followed by Western-blotting and Coomassie blue staining analyses. For Western-blotting analysis, the proteins were transferred to PVDF membrane (Cat. No. IPVH00010; Millipore Sigma). The PVDF membrane was then washed with TBST buffer and blocked with 5% nonfat dried milk (Cat. No. M0841; LabScientific, Highlands, NJ) for 2 h at room temperature, followed by incubation with mouse anti-HIS antibody (1:1000; Cat. No. ab18184; Abcam) at 4 °C overnight. The blot was then washed three times with TBST buffer and incubated with horseradish peroxidase (HRP)-conjugated goat anti-mouse IgG (1:2000; Cat. No. 62-6520; Invitrogen) for 1 h and again washed three times for 10 min with TBST buffer. The PVDF blot was incubated with Bio-Rad clarity Western ECL substrate (Cat. No. 1705060; Bio-Rad), and was imaged using a CL-XPosure film (Cat. No. XAR ALF 1318; LabScientific, Highlands, NJ). For Coomassie blue staining, the SDS-PAGE gel was stained with Coomassie Brilliant Blue R-250 (Cat. No. 1610400; Bio-Rad).

### Mass spectrometry

Mass spectrometry (MS) was performed to determine if the full-length FAM20A was purified by IMAC. The methods for this have been previously described^33^. Briefly, can FAM20A-HIS protein band was carefully excised from the Coomassie blue-stained SDS-PAGE gel and digested overnight with trypsin (Pierce) following reduction and alkylation with DTT and iodoacetamide (Sigma–Aldrich). The samples then underwent solid-phase extraction cleanup with an Oasis HLB plate (Waters) and the resulting samples were injected onto an Orbitrap Fusion Lumos mass spectrometer coupled to an Ultimate 3000 RSLC-Nano liquid chromatography system. Samples were injected onto a 75 um i.d., 75-cm long EasySpray column (Thermo) and eluted with a gradient from 1 to 28% buffer B over 90 min. Buffer A contained 2% (v/canACN and 0.1% formic acid in water, and buffer B contained 80% canv) ACN, 10% (v/v) trifluoroethanol, and 0.1% formic acid in water. The mass spectrometer operated in positive ion mode with a source voltage of 2.0 kV and an ion transfer tube temperature of 275 °C. MS scans were acquired at 120,000 resolution in the Orbitrap and up to 10 MS/MS spectra were obtained in the ion trap for each full spectrum acquired using higher-energy collisional dissociation (HCD) for ions with charges 2–7. Dynamic exclusion was set for 25 s after an ion was selected for fragmentation. Raw MS data files were analyzed using Proteome Discoverer v2.4 SP1 (Thermo), with peptide identification performed using Sequest HT searching against the mouse reviewed protein database from UniProt. Fragment and precursor tolerances of 10 ppm and 0.6 Da were specified, and three missed cleavages were allowed. Loss of N-terminal methionine and N-terminal acetylation were set as protein variable modifications, carbamidomethylation of Cys was set as a fixed peptide modification, and oxidation of Met was set as a peptide variable modification. The false-discovery rate (FDR) cutoff was 1% for all peptides.

### Cell total membrane fractionation and solubilization

Cell total membrane fractionation and solubilization was achieved using Mem-PER™ Plus membrane protein extraction kit (Cat. No. 89842; ThermoFisher Scientific, Rockford, IL, USA). Forty-eight hours after transfection, HEK293 cells transfected with the FAM20A-HIS-expressing construct were resuspend in the growth media by scrapping the cells using a cell scraper. The cells were centrifuged at 300×*g* for 5 min and the supernatant was discarded. The cells were again resuspended in cell wash solution and centrifuged at 300×*g* for 5 min and supernatant was discarded. The cell pellet was then incubated with permeabilization buffer and vortexed briefly to get a homogeneous cell suspension. The suspension was incubated for 10 min at 4 °C with constant mixing. The cells were then centrifuged for 15 min at 16,000×*g* and the supernatant containing soluble proteins was carefully transferred to a new tube. The pellet (total membranes) was then resuspended in membrane solubilization buffer and incubated at 4 °C for 30 min with constant mixing. The cells were again centrifuged at 16,000×*g* for 15 min at 4 °C. The supernatant containing membrane and membrane linked proteins was transferred for downstream processing. Membrane and soluble proteins were mixed with a Laemmli buffer (Cat. No. 1610747; Bio-Rad), and were subject to Western-blotting analysis as described above. Briefly, once the proteins were transferred to the PVDF membrane, the PVDF membrane was sequentially probed with three primary antibodies, mouse anti-HIS antibody (1:1000; Abcam, USA), mouse anti-GAPDH antibody (1:2000; Cat. No. ab8245; Abcam, USA), and rabbit anti-sodium potassium ATPase antibody (1:10,000; Cat. No. ab76020; Abcam, USA), followed by incubation with HRP-conjugated goat anti-mouse IgG (H + L) (Cat. No. 62-6520; Invitrogen) or goat anti-rabbit IgG (H + L) secondary antibody (Cat. No. 65-6120; Invitrogen), respectively.

### Western-blotting analysis of FAM20A secretion

HEK293 cells in a 6-well plate were transiently transfected with a total of 3 µg of pCDNA3 empty vector or a construct expressing FAM20A-FLAG and/or FAM20C-FLAG. On the next day, the transfection medium was replaced with 1.5 ml serum-free DMEM. The cells were further cultured for 48 h, and the total cell lysate and conditioned medium were harvested and analyzed by Western-blotting as described above. Briefly, after the proteins were transferred to a PVDF membrane, the PVDF membrane was incubated with mouse monoclonal anti-FLAG M2 antibody (1:500; Cat. No. F1804; Sigma), and the blot was then stripped and probed with mouse monoclonal β-actin antibody (1:20, 000; Cat. No. A3854; Sigma). Conditioned medium was concentrated, if needed, using Amicon Ultra-0.5 centrifugal filter unit with a cutoff of 10 kDa (Cat. No. UFC501096; Millipore Sigma).

### Co-immunofluorescent staining

Co-immunofluorescent staining was performed to determine the subcellular localization of FAM20A in 17IIA11 and LS8 cells, as described previously^34^. Briefly, transfected and non-transfected 17IIA11 and LS8 cells were fixed with 4% paraformaldehyde (PFA), and then selectively permeabilized with 12.5 µM digitonin (Cat. No. 300410; Sigma, USA) or completely permeabilized with 0.1% Triton X-100 (Cat. No. T8532; Sigma, USA). The cells were subsequently blocked with a blocking buffer containing 1% goat serum (Cat. No. 642921; MP Biomedicals) in 1xPBS for 2 h at room temperature, and incubated with the primary antibodies diluted in the blocking buffer at 4 °C overnight. The primary antibodies used in this study included mouse-monoclonal anti-FLAG M2 antibody (1:1000; Sigma, USA), rabbit anti-FAM20A antibody (1:250; Cat. No: A8496; ABclonal), rabbit-anti-GM130 antibody (1:1000; Cat. No. ab52649; Abcam, USA), mouse monoclonal anti-GM130 antibody (1:1000; Cat. No.: NBP3-23353; Novus Biological, USA), and rabbit-polyclonal anti-Calnexin antibody (1:1000; Cat. No. ab22595; Abcam, USA). The cells were then incubated with secondary antibodies, a combination of Alexa Fluor™ 594-conjugated goat anti-mouse IgG (H + L) cross-adsorbed secondary antibody (1:1000; Cat. No. A-11005; ThermoFisher Scientific) and Alexa Fluor™ 488-conjugated goat anti-rabbit IgG (H + L) cross-adsorbed secondary antibody (1:1000; Cat. No. A-11008; ThermoFisher Scientific) or a combination of Alexa Fluor™ 594-conjugated goat anti-rabbit IgG (H + L) cross-adsorbed secondary antibody (1:1000; Cat. No. A-11012; Invitrogen) and Alexa Fluor™ 488-conjugated goat anti-mouse IgG (H + L) cross-adsorbed secondary antibody (1:1000; Cat. No. A-11001; Invitrogen) for 1 h at room temperature in the dark. The glass slides were mounted using ProLong™ diamond antifade mountant with 4′, 6-diamidino-2-phenylindole (DAPI) (Cat. No. P36962; ThermoFisher Scientific, USA). Glass coverslips were viewed and imaged under Leica DM4 B upright fluorescence microscope equipped with a K5 sCMOS camera (Leica Microsystem).

### Immunohistochemistry

Immunohistochemistry was conducted to determine the subcellular localization of FAM20A in odontoblasts and ameloblasts in mice, as previously described^35^. Following euthanasia with CO_2_, the mandibles of 4-day-old wild-type mice were dissected and fixed in 4% PFA in PBS at 4 °C overnight, and were decalcified with 15% disodium ethylenediaminetetraacetic acid (EDTA, pH 7.4) for 4 days on a shaker. The decalcified mandibles were then dehydrated through a series of gradient ethanol, cleared by xylene, embedded in paraffin, and were cut into serial sagittal sections at a thickness of 5 µm. Sections were collected on Fisherbrand Superfrost Plus Microscope Slides (Cat. No. 12-550-15; Fisher Scientific). For immunohistochemical analysis, the paraffin sections were heated at 60 °C for 1 h, dewaxed in xylene, and then rehydrated through a series of gradient ethanol. Antigen retrieval was performed by incubating the slides in Tris–EDTA buffer (10 mM Tris base, 1 mM EDTA, 0.05% Tween 20, pH 9.0) (Leica Retrieval Solution pH 9.0; Cat. No. RE7119; Leica Biosystems) in a jar placed within an IHC-Tek Epitope retrieval steamer for 15 min. The sections were washed with PBST (0.1% Tween 20 in 1X PBS) and permeabilized with 0.2% Triton X-100 (Sigma, USA) for 20 min. The sections were again washed with PBST and blocked with blocking buffer containing 3% bovine serum albumin, 10% goat serum (Cat. No. 642921; MP Biomedicals) in PBST at room temperature for 2 h in a humidified chamber. The sections were then incubated with the primary antibodies including a rabbit anti-FAM20A antibody (1:250; ABclonal) and a mouse monoclonal anti-GM130 antibody (1:500; Novus Biological, USA) diluted in blocking buffer at room temperature for 1 h, and then at 4 °C overnight. The sections were washed with PBST and incubated with the secondary antibodies including an Alexa Fluor™ 594-conjugated goat anti-rabbit IgG (H + L) cross-adsorbed secondary antibody (1:1000; Invitrogen) and Alexa Fluor™ 488-conjugated goat anti-mouse IgG (H + L) cross-adsorbed secondary antibody (1:1000; Invitrogen) for 1 h at room temperature in the dark. The glass slides were washed, mounted and imaged as described above.

### Supplementary Information

Supplementary Information.

## Data Availability

All data generated or analyzed during this study are included in this published article (and its Supplementary Information files).
