# Supplementary material for: FAM20A is a golgi-localized Type II transmembrane protein
Source: Sci Rep. 2024 Mar 18;14:6518. doi: 10.1038/s41598-024-57007-z (PMC10948845; doi:10.1038/s41598-024-57007-z)
Supplement: Supplementary file 1 — Supplementary Information. [file 41598_2024_57007_MOESM1_ESM.pdf]

FAM20A is a Golgi-localized Type II transmembrane protein

Mohammad Faizan Siddiqui, Jiahe Li, Suzhen Wang, Hua Zhang, Chunlin Qin, Yongbo Lu

Department of Biomedical Sciences, Texas A&M University School of Dentistry, Dallas, TX, USA 75246

Supplementary information

Materials and methods

Table S1 The peptides identified by mass spectrometry from tryptic digests of FAM20A gel band.

Figure S1 Western-blotting and Coomassie blue staining analyses of purified FAM20A.

Figure S2 Membrane localization of FAM20A.

Figure S3 Signal peptide and membrane topology of FAM20A predicted by TOPCONS.

Figure S4 Signal peptide and membrane topology of FAM20B predicted by TOPCONS.

Figure S5 Signal peptide and membrane topology of FAM20C predicted by TOPCONS.

Materials and methods

Topology prediction of FAM20 members

TOPCONS is a web server (https://topcons.cbr.su.se/) for prediction of membrane protein topology and signal peptide [1]. The TOPCONS Hidden Markov Model (HMM) uses predictions generated by these five different algorithms, OCTOPUS [2], Philius [3], PolyPhobius [4], SCAMPI [5], and SPOCTOPUS [6] as inputs and generates a consensus prediction for the protein of interest along with a reliability score based on the agreement of the included methods across the sequence. To determine if the N-terminal region of each FAM20 family member functions as a signal/anchor sequence, the amino acid sequence of FAM20A (Mus musculus; accession number: Q8CID3), FAM20B and FAM20C was submitted to the TOPCONS web server for prediction of signal peptide and transmembrane helix.

References

1 Tsirigos, K. D., Peters, C., Shu, N., Kall, L. & Elofsson, A. The TOPCONS web server for consensus prediction of membrane protein topology and signal peptides. *Nucleic Acids Res* **43**, W401-407, doi:10.1093/nar/gkv485 (2015).

2 Viklund, H. & Elofsson, A. OCTOPUS: improving topology prediction by two-track ANN-based preference scores and an extended topological grammar. *Bioinformatics* **24**, 1662-1668, doi:10.1093/bioinformatics/btn221 (2008).

3 Reynolds, S. M., Kall, L., Riffle, M. E., Bilmes, J. A. & Noble, W. S. Transmembrane topology and signal peptide prediction using dynamic bayesian networks. *PLoS Comput Biol* **4**, e1000213, doi:10.1371/journal.pcbi.1000213 (2008).

4 Kall, L., Krogh, A. & Sonnhammer, E. L. An HMM posterior decoder for sequence feature prediction that includes homology information. *Bioinformatics* **21 Suppl 1**, i251-257, doi:10.1093/bioinformatics/bti1014 (2005).

5 Bernsel, A. *et al.* Prediction of membrane-protein topology from first principles. *Proc Natl Acad Sci U S A* **105**, 7177-7181, doi:10.1073/pnas.0711151105 (2008).

6 Viklund, H., Bernsel, A., Skwark, M. & Elofsson, A. SPOCTOPUS: a combined predictor of signal peptides and membrane protein topology. *Bioinformatics* **24**, 2928-2929, doi:10.1093/bioinformatics/btn550 (2008).

Table S1 The peptides identified by mass spectrometry from tryptic digests of FAM20A gel band

Confidence	Annotated Sequence	Modifications	[a]PSMs	Positions in Master Proteins	[b]Abundance
High	[-].MPGLRRDRLLALLLLGALFSADLYFHLWPQVQR.[Q]	1xOxidation [M1]	1	Q8CID3 [1-33]	7.31E+04
High	[R].RDRLLALLLLGALFSADLYFHLWPQVQR.[Q]		2	Q8CID3 [6-33]	9.90E+04
High	[R].DRLLALLLLGALFSADLYFHLWPQVQR.[Q]		2	Q8CID3 [7-33]	5.97E+06
High	[R].LLALLLLGALFSADLYFHLWPQVQR.[Q]		2	Q8CID3 [9-33]	9.87E+05
High	[R].QLRPGERPAACPCSGR.[A]	2xCarbamidomethyl [C11; C13]	6	Q8CID3 [34-49]	3.11E+06
High	[R].APSASLHSAAASR.[D]		47	Q8CID3 [50-62]	2.72E+08
High	[R].DLGTASHNFSGALPR.[V]		5	Q8CID3 [63-77]	2.66E+05
High	[R].SKLQALFAHSLYQVLEDPPLLGPEDWLLASQEALR.[Y]		2	Q8CID3 [90-124]	6.56E+05
High	[K].LQALFAHSLYQVLEDPPLLGPEDWLLASQEALR.[Y]		2	Q8CID3 [92-124]	6.51E+06
High	[R].HFPTISADYSQDEK.[A]		20	Q8CID3 [190-203]	2.73E+07
High	[K].ALLGACDCSQIVKPSGVHLK.[L]	2xCarbamidomethyl [C6; C8]	15	Q8CID3 [204-223]	8.47E+06
High	[K].AMFKPMR.[Q]	2xOxidation [M2; M6]	5	Q8CID3 [234-240]	4.22E+06
High	[R].QQREEETPEDFFYFIDFQR.[H]		3	Q8CID3 [241-259]	4.12E+06
High	[R].EEETPEDFFYFIDFQR.[H]		3	Q8CID3 [244-259]	1.01E+07
High	[R].HNAEIAAFHLDR.[I]		9	Q8CID3 [260-271]	2.81E+06
High	[R].RVPPTVGR.[L]		42	Q8CID3 [277-284]	9.17E+07
High	[K].EILEVTKNEILQSVFFVSPANNVCFFAK.[C]	1xCarbamidomethyl [C24]	1	Q8CID3 [291-318]	7.03E+05
High	[K].EILEVTK.[N]		6	Q8CID3 [291-297]	2.10E+07
High	[K].NEILQSVFFVSPANNVCFFAK.[C]	1xCarbamidomethyl [C17]	1	Q8CID3 [298-318]	2.52E+06
High	[K].CPYMCK.[T]	2xCarbamidomethyl [C1; C5]. 1xOxidation [M4]	28	Q8CID3 [319-324]	6.32E+07
High	[K].CPYMCK.[T]	2xCarbamidomethyl [C1; C5]	1	Q8CID3 [319-324]	6.30E+06
High	[K].TEYAVCGNPHLLEGSLSAFLPSLNLAPR.[L]	1xCarbamidomethyl [C6]	1	Q8CID3 [325-352]	3.39E+06
High	[R].LSVPNPWIR.[S]		19	Q8CID3 [353-361]	2.61E+07
High	[R].SYSLSGKEEWELNPLYCDTVK.[Q]	1xCarbamidomethyl [C17]	8	Q8CID3 [362-382]	1.05E+06
High	[K].EEWELNPLYCDTVK.[Q]	1xCarbamidomethyl [C10]	8	Q8CID3 [369-382]	9.41E+05
High	[K].QIYPYNSSNR.[L]		40	Q8CID3 [383-392]	3.89E+07
High	[R].LLGIIDMAVFDFLIGNMDR.[H]	1xOxidation [M17]	1	Q8CID3 [393-411]	1.66E+05
High	[R].LLGIIDMAVFDFLIGNMDR.[H]	2xOxidation [M7; M17]	3	Q8CID3 [393-411]	1.52E+06
High	[R].LLGIIDMAVFDFLIGNMDRHHYEMFTK.[F]	3xOxidation [M7; M17; M24]	1	Q8CID3 [393-419]	7.97E+05
High	[R].HHYEMFTK.[F]	1xOxidation [M5]	1	Q8CID3 [412-419]	3.81E+05
High	[K].FGDDGYLIHLDNAR.[G]		3	Q8CID3 [420-433]	2.73E+06
High	[R].HSQDEISILAPLAQCCMIK.[R]	2xCarbamidomethyl [C15; C16]	2	Q8CID3 [438-456]	3.35E+05
High	[R].HSQDEISILAPLAQCCMIK.[R]	2xCarbamidomethyl [C15; C16]; 1xOxidation [M17]	12	Q8CID3 [438-456]	1.53E+07
High	[R].HSQDEISILAPLAQCCMIKR.[K]	2xCarbamidomethyl [C15; C16]; 1xOxidation [M17]	1	Q8CID3 [438-457]	
High	[R].KTLLHLQLLAQADYR.[L]		2	Q8CID3 [458-472]	2.20E+05
High	[K].TLLHLQLLAQADYR.[L]		7	Q8CID3 [459-472]	2.39E+06
High	[R].LSDVMRESLLEDQLSPVLTEPHLLALDR.[R]	1xOxidation [M5]	1	Q8CID3 [473-500]	1.55E+05
High	[R].LSDVMRESLLEDQLSPVLTEPHLLALDRR.[L]	1xOxidation [M5]	1	Q8CID3 [473-501]	5.99E+04
High	[R].ESLLEDQLSPVLTEPHLLALDR.[R]		5	Q8CID3 [479-500]	3.14E+07
High	[R].ESLLEDQLSPVLTEPHLLALDRR.[L]		2	Q8CID3 [479-501]	1.13E+06
High	[RK].RLQIILK.[TG]		3	Q8CID3 [501-507]	7.23E+06
High	[K].TVEDCIEAHGER.[R]	1xCarbamidomethyl [C5]	43	Q8CID3 [508-519]	2.77E+07
High	[K].TVEDCIEAHGERR.[V]	1xCarbamidomethyl [C5]	2	Q8CID3 [508-520]	1.97E+06

High	[R].RVIAEGSAQR.[S]		26	Q8CID3 [520-529]	1.09E+07
High	[R].VIAEGSAQR.[S]		63	Q8CID3 [521-529]	3.93E+08

Notes: [a], the number of peptide spectrum matches, or the number of spectra assigned to peptides that contributed to the inference of the protein.
[b], the sum of the peak intensity for each peptide identified for that protein.

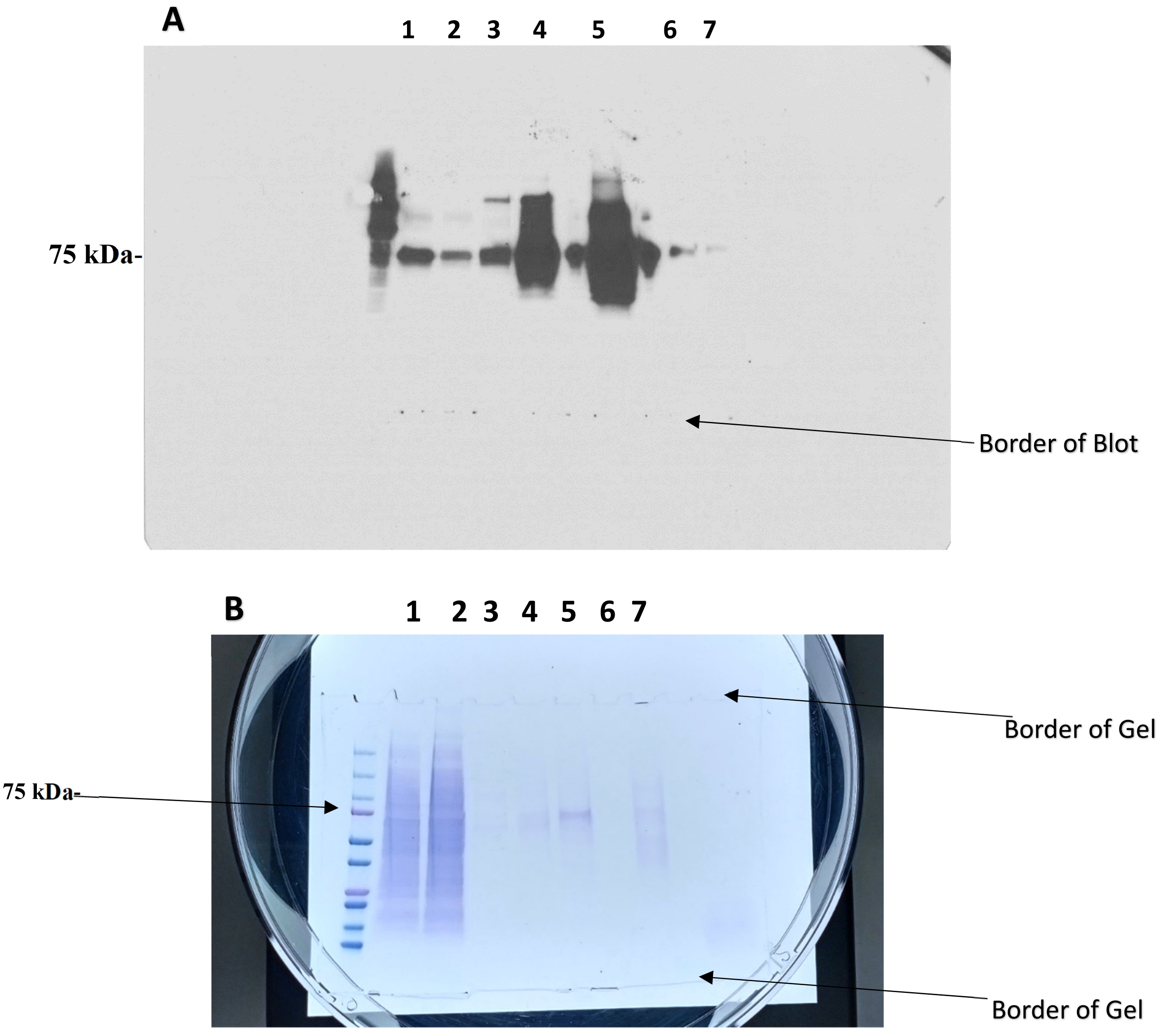

Figure S1. **Western-blotting and Coomassie blue staining analyses of purified FAM20A.** The fractions eluted from IMAC were analyzed by Western-blotting analysis using an anti-HIS antibody for detection of FAM20A-HIS (**A**), and by Coomassie blue staining of SDS-PAGE gel (**B**). Lane 1 is the cell lysate; lane 2 is the flow-through; and lanes 3 through 7 are the fractions eluted with 50, 100, 250, 500, and 1000 mM imidazole, respectively. (original blot and gel)

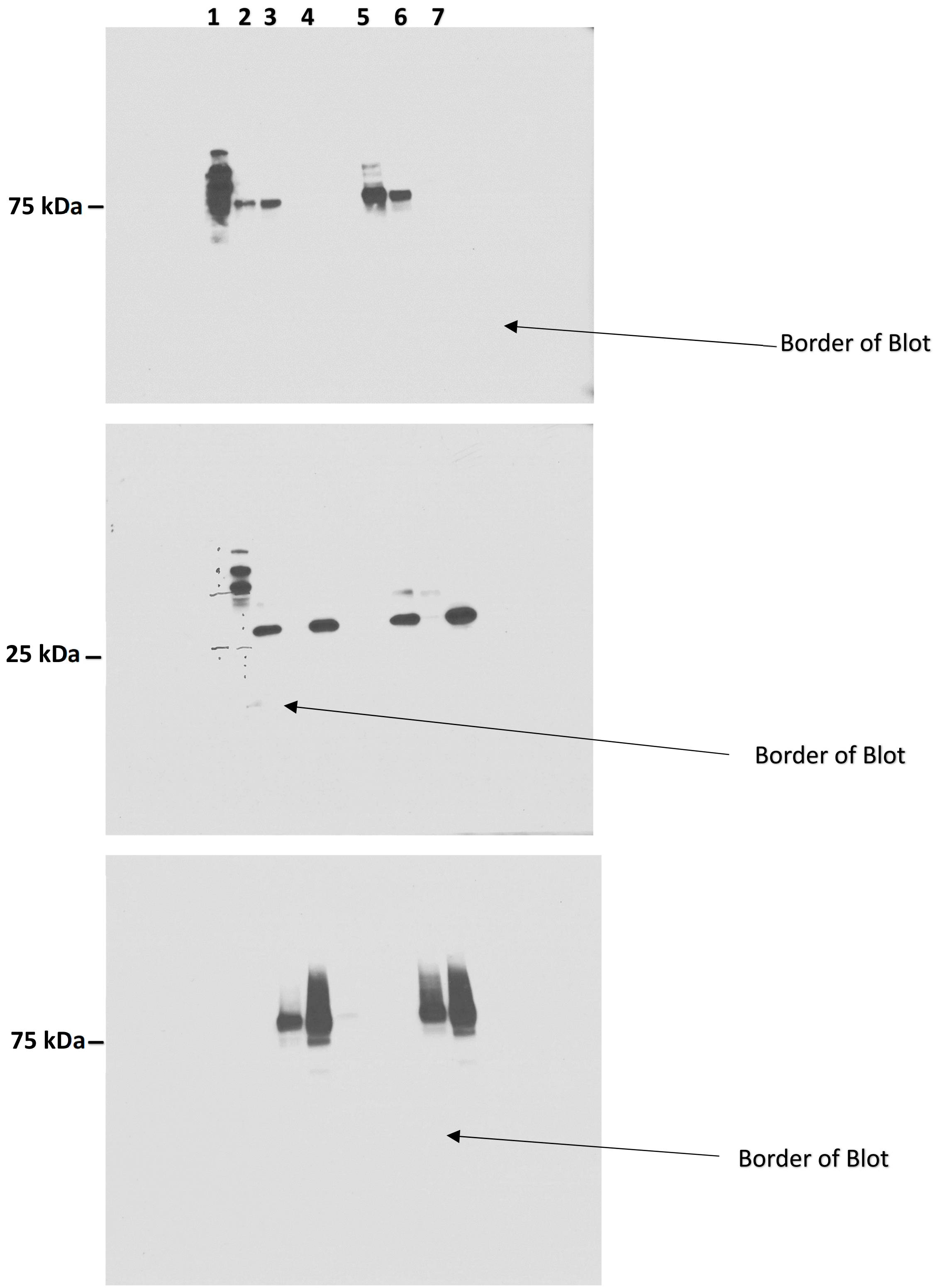
1 2 3 4 5 6 7
75 kDa
Border of Blot
25 kDa
Border of Blot
75 kDa
Border of Blot

Figure S2 Membrane localization of FAM20A. A. FAM20A present in the membrane fraction of the expressing cells. Western-blotting analysis of FAM20A in the membrane and soluble fractions extracted from HEK293 cells transiently transfected with the construct expressing FAM20A-HIS. The blot was first probed with an anti-HIS antibody against FAM20A-HIS (top panel). The blot was then sequentially probed with an anti-GAPDH (middle panel) and anti-sodium potassium ($Na^{+}K^{+}$) ATPase antibody (bottom panel). GAPDH and sodium potassium ATPase were used as a cytosolic protein marker and plasma membrane protein marker, respectively. L, total cell lysate; M, membrane fraction; and S, soluble fraction. **Notes: Lanes 5, 6 and 7 shown in the manuscript.** (original blots)

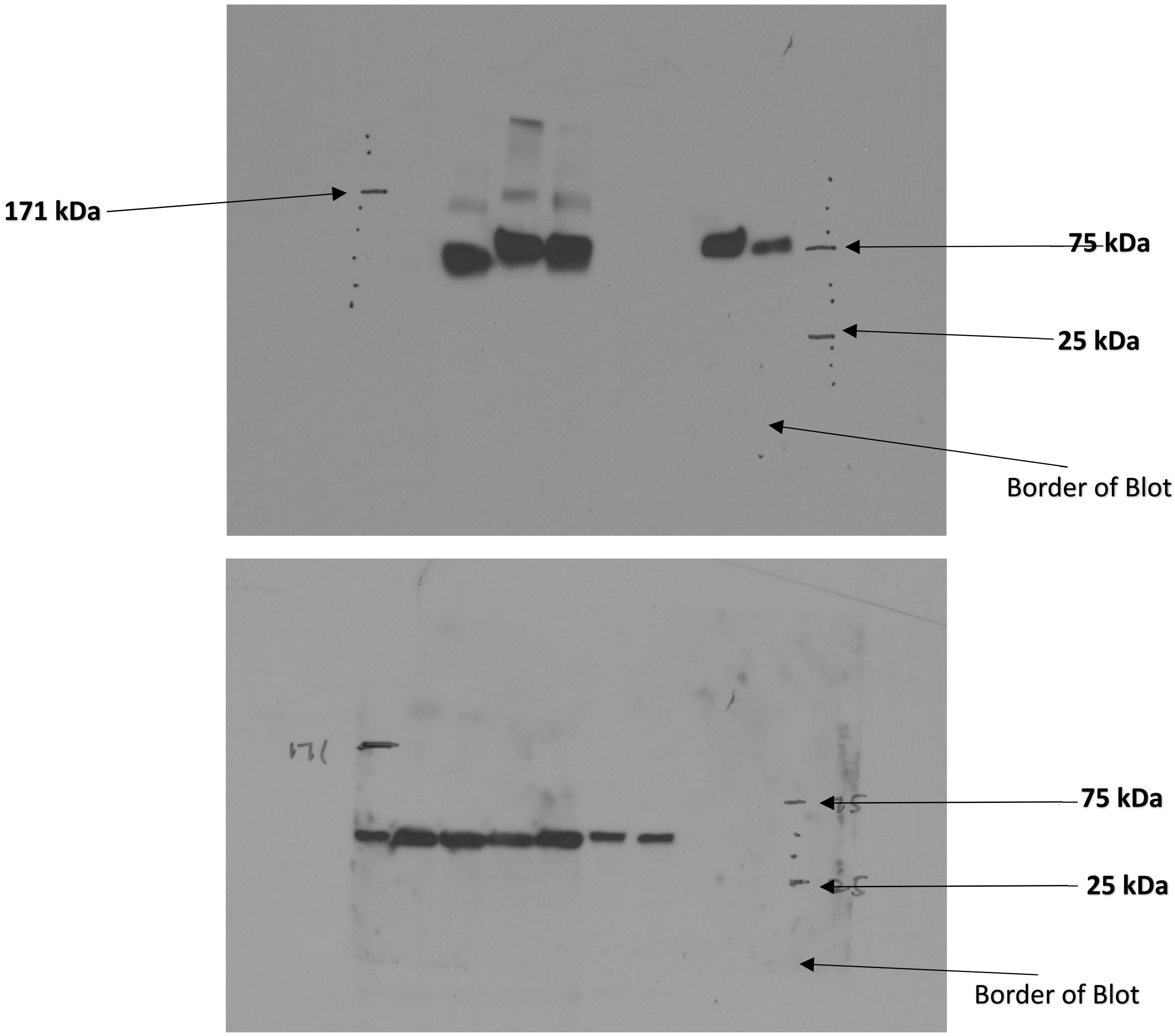

Figure S2 B. FAM20A is not secreted out the expressing cells. HEK293 cells were transiently transfected with pCDNA3 empty vector (Vector) or a construct expressing FAM20A-FLAG and/or FAM20C-FLAG. The total cell lysates and conditioned media were harvested and analyzed by Western-blotting with a mouse monoclonal anti-FLAG M2 antibody (top panel), and the blot was then stripped and probed with a mouse monoclonal β-actin antibody (bottom panel). The amount of DNA transfected for each construct are indicated. For lanes 1, 2 and 3, 60 µg of total cell lysates were loaded. For lanes 5 and 6, 1 ml of conditioned medium was loaded after concentration; and for lanes 7 and 8, 20 µl of conditioned medium was loaded. Note that β-actin was detectable in lanes 5 and 6 loaded with concentrated medium. (original blots)

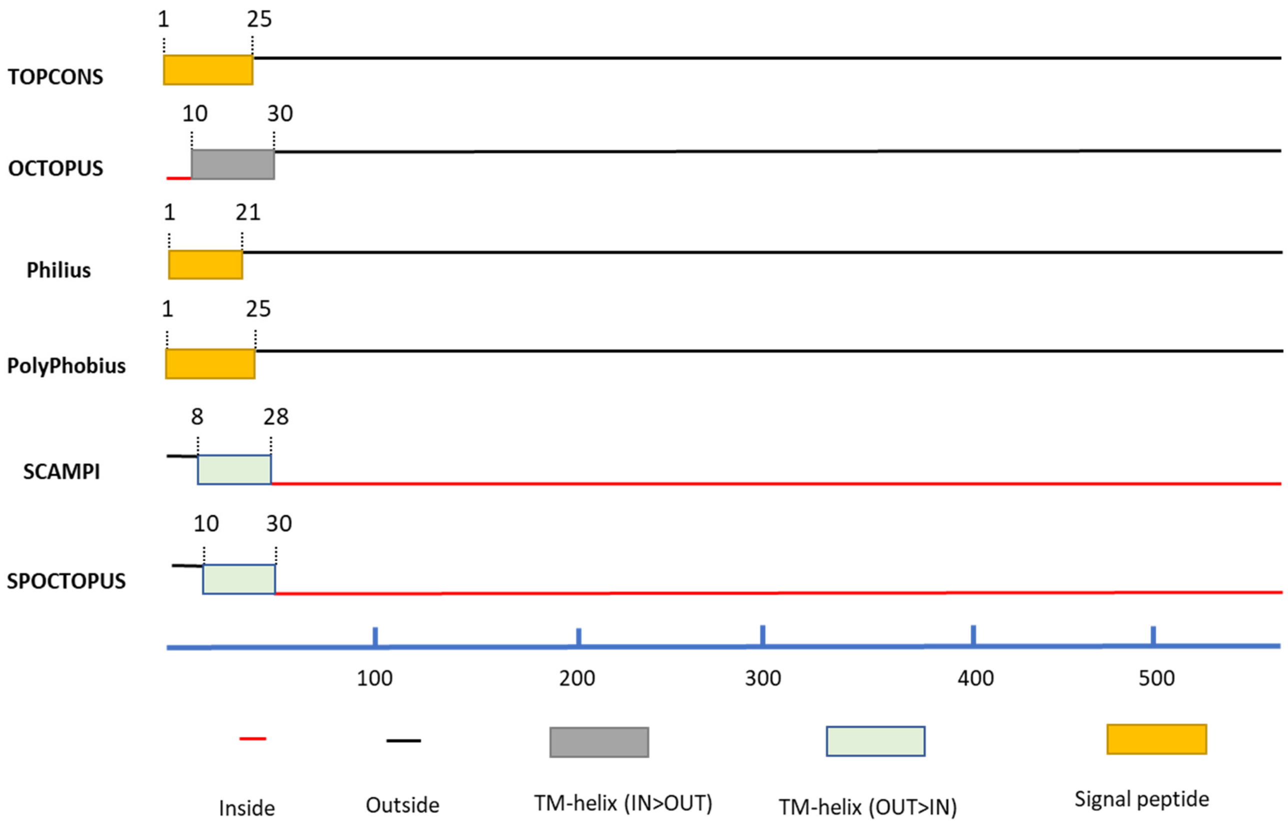

Figure S3 Signal peptide and membrane topology of FAM20A predicted by TOPCONS. The amino acid sequence of FAM20A (Mus musculus; accession number: Q8CID3) was submitted to TOPCONS for prediction of signal peptide (SP) and transmembrane (TM) helix. TOPCONS: SP (1-25); OCTOPUS: TM1 (10-30); PHILIUS: SP (1-21); POLYPHOBIUS: SP (1-25); SCAMPI: TM1 (8-28); and SPOCTOPUS: TM1 (10-30). All numbers refer to the amino acid sequence of FAM20A starting from 1. Inside, inside the membrane; outside, outside the membrane.

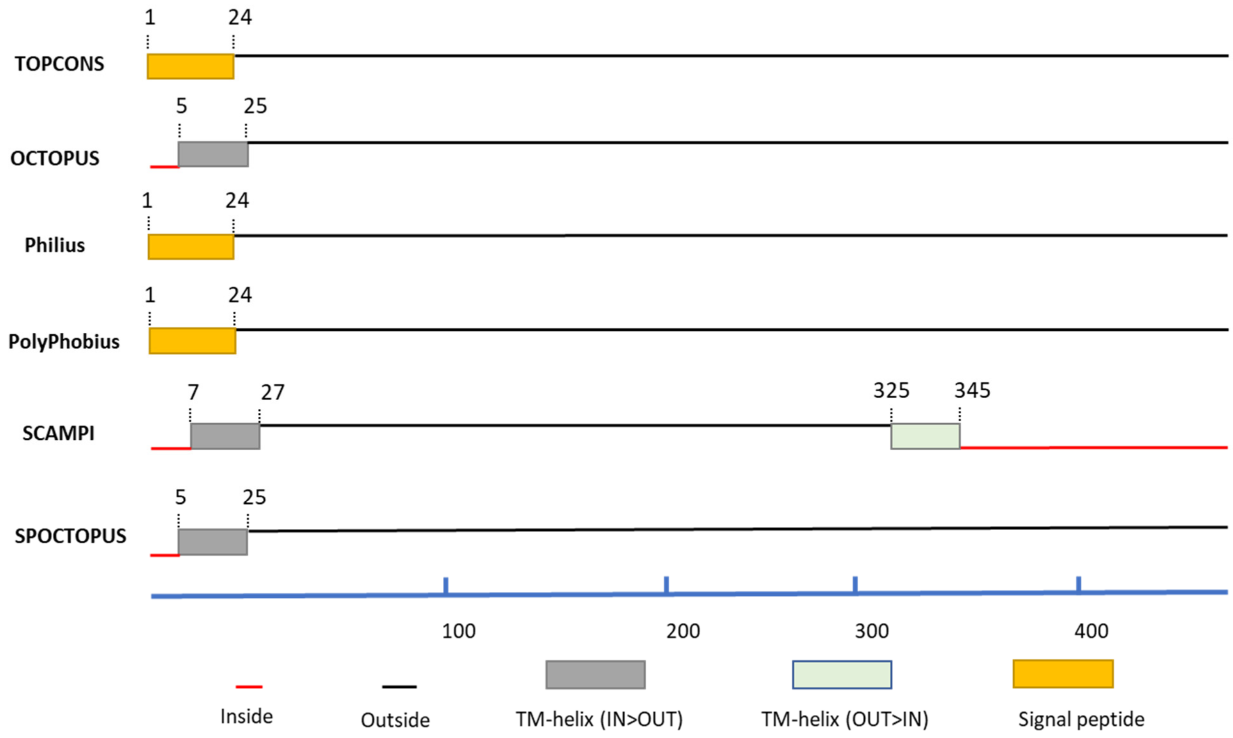

Figure S4 Signal peptide and membrane topology of FAM20B predicted by TOPCONS. The amino acid sequence of FAM20B (Mus musculus; accession number: Q8VCS3) was submitted to TOPCONS for prediction of signal peptide (SP) and transmembrane (TM) helix. TOPCONS: SP (1-24); OCTOPUS: TM1 (5-25); Philius: SP (1-24); PolyPhobius: SP (1-24); SCAMPI: TM1 (7-27) and TM2 (325-345); and SPOCTOPUS: TM1 (5-25). All numbers refer to the amino acid sequence of FAM20B starting from 1. Inside, inside the membrane; outside, outside the membrane.

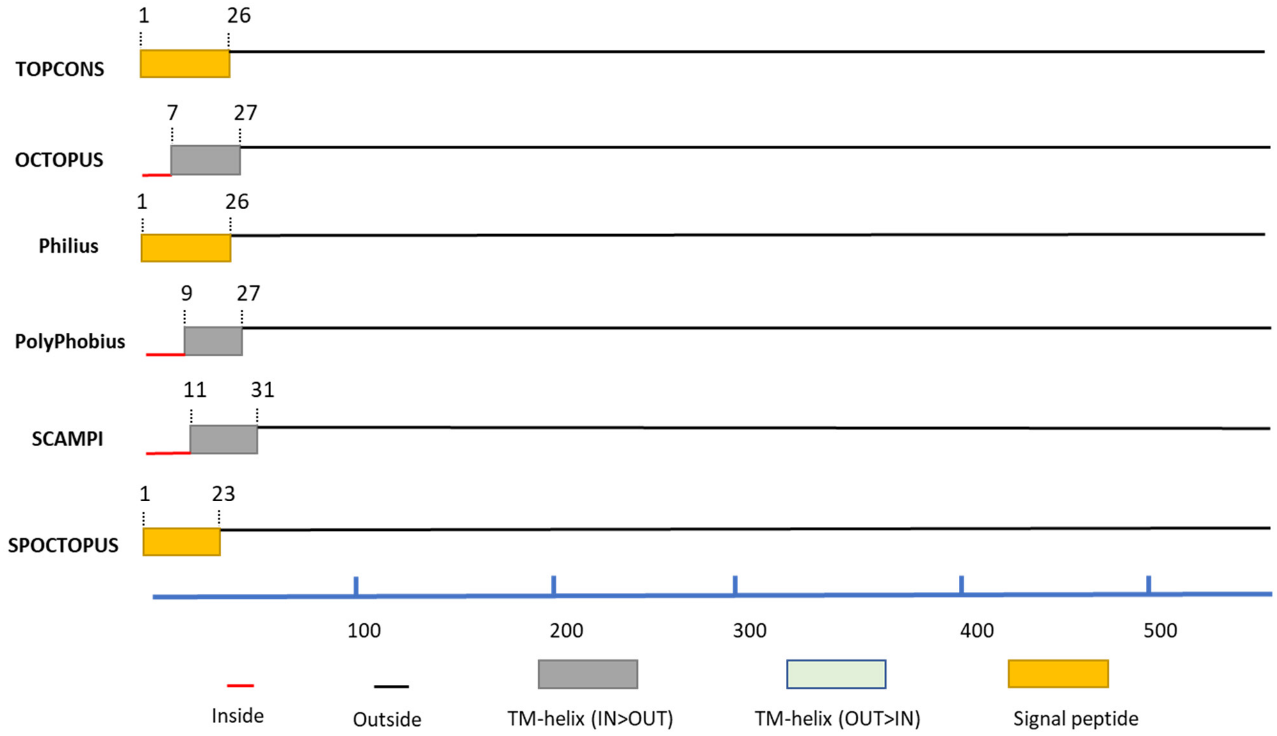

Figure S5 Signal peptide and membrane topology of FAM20C predicted by TOPCONS. The amino acid sequence of FAM20C (Mus musculus; accession number: Q5MJS3) was submitted to TOPCONS for prediction of signal peptide (SP) and transmembrane (TM) helix. TOPCONS: SP (1-26); OCTOPUS: TM1 (7-27); Philius: SP (1-26); PolyPhobius: TM1 (9-27); SCAMPI: TM1 (11-31); and SPOCTOPUS: SP (1-23). All numbers refer to the amino acid sequence of FAM20C starting from 1. Inside, inside the membrane; outside, outside the membrane.
